# High Bone Mass Disorders: New Insights From Connecting the Clinic and the Bench

**DOI:** 10.1002/jbmr.4715

**Published:** 2022-10-21

**Authors:** Dylan J.M. Bergen, Antonio Maurizi, Melissa M. Formosa, Georgina L.K. McDonald, Ahmed El‐Gazzar, Neelam Hassan, Maria‐Luisa Brandi, José A. Riancho, Fernando Rivadeneira, Evangelia Ntzani, Emma L. Duncan, Celia L. Gregson, Douglas P. Kiel, M. Carola Zillikens, Luca Sangiorgi, Wolfgang Högler, Ivan Duran, Outi Mäkitie, Wim Van Hul, Gretl Hendrickx

**Affiliations:** ^1^ School of Physiology, Pharmacology, and Neuroscience, Faculty of Life Sciences University of Bristol Bristol UK; ^2^ Musculoskeletal Research Unit, Translational Health Sciences, Bristol Medical School, Faculty of Health Sciences University of Bristol Bristol UK; ^3^ Department of Biotechnological and Applied Clinical Sciences University of L'Aquila L'Aquila Italy; ^4^ Department of Applied Biomedical Science, Faculty of Health Sciences University of Malta Msida Malta; ^5^ Center for Molecular Medicine and Biobanking University of Malta Msida Malta; ^6^ Department of Paediatrics and Adolescent Medicine Johannes Kepler University Linz Linz Austria; ^7^ Italian Bone Disease Research Foundation (FIRMO) Florence Italy; ^8^ Department of Internal Medicine, Hospital U M Valdecilla University of Cantabria, IDIVAL Santander Spain; ^9^ Department of Internal Medicine Erasmus University Medical Center Rotterdam The Netherlands; ^10^ Department of Hygiene and Epidemiology, Medical School University of Ioannina Ioannina Greece; ^11^ Center for Evidence Synthesis in Health, Policy and Practice, Center for Research Synthesis in Health, School of Public Health Brown University Providence RI USA; ^12^ Institute of Biosciences University Research Center of loannina, University of Ioannina Ioannina Greece; ^13^ Department of Twin Research & Genetic Epidemiology, School of Life Course Sciences, Faculty of Life Sciences and Medicine King's College London London UK; ^14^ Department of Endocrinology Guy's and St Thomas' NHS Foundation Trust London UK; ^15^ Marcus Institute for Aging Research, Hebrew SeniorLife and Department of Medicine Beth Israel Deaconess Medical Center and Harvard Medical School Broad Institute of MIT & Harvard Cambridge MA USA; ^16^ Department of Rare Skeletal Diseases IRCCS Rizzoli Orthopaedic Institute Bologna Italy; ^17^ Institute of Metabolism and Systems Research University of Birmingham Birmingham UK; ^18^ University of Malaga Malaga Spain; ^19^ Children's Hospital University of Helsinki and Helsinki University Hospital Helsinki Finland; ^20^ Research Program for Clinical and Molecular Metabolism, Faculty of Medicine University of Helsinki Helsinki Finland; ^21^ Folkhälsan Research Centre Folkhälsan Institute of Genetics Helsinki Finland; ^22^ Department of Medical Genetics University of Antwerp Antwerp Belgium; ^23^ Department of Human Genetics KU Leuven Leuven Belgium

**Keywords:** DISEASES AND DISORDERS OF/RELATED TO BONE, ANABOLICS, THERAPEUTICS, GENETIC ANIMAL MODELS, ANIMAL MODELS, CELL/TISSUE SIGNALING, PARACRINE PATHWAYS, GENETIC RESEARCH

## Abstract

Monogenic high bone mass (HBM) disorders are characterized by an increased amount of bone in general, or at specific sites in the skeleton. Here, we describe 59 HBM disorders with 50 known disease‐causing genes from the literature, and we provide an overview of the signaling pathways and mechanisms involved in the pathogenesis of these disorders. Based on this, we classify the known HBM genes into HBM (sub)groups according to uniform Gene Ontology (GO) terminology. This classification system may aid in hypothesis generation, for both wet lab experimental design and clinical genetic screening strategies. We discuss how functional genomics can shape discovery of novel HBM genes and/or mechanisms in the future, through implementation of omics assessments in existing and future model systems. Finally, we address strategies to improve gene identification in unsolved HBM cases and highlight the importance for cross‐laboratory collaborations encompassing multidisciplinary efforts to transfer knowledge generated at the bench to the clinic. © 2022 The Authors. *Journal of Bone and Mineral Research* published by Wiley Periodicals LLC on behalf of American Society for Bone and Mineral Research (ASBMR).

## Introduction

The lifelong dynamics of bone health depend on the bone remodeling cycle, where a continuous interplay between age‐related, environmental and genetic risk factors affect the metabolic activity of bone building cells (osteoblasts) and bone degrading cells (osteoclasts).^(^ ^1^ ^)^ In a healthy setting, the metabolic equilibrium of bone anabolism and catabolism results in the preservation of a mineralized organic matrix. When this balance is disrupted, individuals are prone to develop disorders with either low bone mass (LBM) or elevated bone mass with or without dense bones, commonly known as high bone mass (HBM). LBM, the most common disorder being osteoporosis, is defined as an areal bone mineral density (aBMD) *T*‐score of ≤ −2.5 at the post‐anterior lumbar spine, hip, radius, or whole body by dual‐energy X‐ray absorptiometry (DXA) scans in postmenopausal women and males older than 50 years, or an aBMD *Z*‐score of ≤ −2.0 in premenopausal women and young adults (<50 years).^(^ ^2^, ^3^, ^4^ ^)^ Monogenic LBM disorders have been reviewed in detail in the first flagship paper published on behalf the GEMSTONE Working Group 3 COST Action.^(^ ^4^ ^)^ In the case of HBM, a net gain of bone mass may often result from a decreased osteoclastic bone resorption, an increased osteoblastic bone formation, and/or a change in the cellular coupling between osteoblasts and osteoclasts favoring anabolism. In this review we focus on genetic disorders of primary HBM that are defined by a generalized increase in *Z*‐score of at least +2.5 in aBMD in at least two skeletal sites by DXA.^(^ ^5^ ^)^

Understanding the clinical and functional features and genetic causes of extreme phenotypes with HBM can improve diagnostics and treatment of patients. Moreover, simultaneously, novel biological drug targets may be discovered, allowing development of new therapies for osteoporosis. A prominent example of such success was the discovery of loss‐of‐function (LoF) mutations in *SOST* encoding sclerostin in families with sclerosteosis (OMIM 269500) and van Buchem disease (OMIM 239100), two severe HBM conditions.^(^ ^6^, ^7^, ^8^ ^)^ A concerted multidisciplinary research effort then unraveled the precise function and effects of sclerostin in the regulation of bone mass, leading to the development of potent osteoporosis therapies; ie, anti‐sclerostin antibodies (eg, romosozumab, blosozumab).^(^ ^9^ ^)^ Over the past few decades, the listing, definition and our knowledge on rare and ultrarare HBM disorders has expanded significantly. Because HBM disorders are multifaceted, this research comprises multiple disciplines, from in‐depth phenotyping and genetic screening of patients to basic wet‐lab science, bringing together molecular and cell biologists, system biologists, and clinician researchers.

In this review, we discuss strategies to advance both clinical genetic knowledge and functional understanding of mechanisms leading to HBM. Similar mechanisms that predispose to secondary or artifactual forms of HBM (eg, osteoarthritis, ankylosing spondylitis, vascular calcification, incidentaloma, etc.) and ectopic bone formation in soft tissues (eg, fibrodysplasia ossificans progressiva [FOP]) are beyond the scope of this review and have recently been reviewed elsewhere.^(^ ^5^, ^10^, ^11^ ^)^ We focus on the mechanisms that underpin the development of monogenic Mendelian HBM disorders. We discuss knowledge collected from functional studies and describe how the HBM field can advance its functional understanding by scrutinizing currently lesser studied mechanisms. Finally, we classify all known HBM genes and their associated disorders according to their role in a signaling pathway or biological process, using uniform Gene Ontology (GO) accession numbers to create HBM (sub)groups.

## Knowledge of Disease Mechanisms Identified in Monogenic Disorders

Most of our knowledge concerning Mendelian, ie, monogenic, HBM disorders and mechanisms has been based on forward genetic approaches. Forward genetics begins with the identification of a HBM phenotype in the clinic, followed by determining the genetic cause of that phenotype and, mostly, functional experiments to confirm the causality of the identified variant.^(^ ^3^, ^4^ ^)^

### Current gene identification strategies

Screening an individual with HBM for pathological variants in the known causative genes is, in many countries, now routine, through the clinical application of high‐throughput sequencing (HTS) (reviewed elsewhere).^(^ ^12^ ^)^ HTS technologies, previously referred to as next generation sequencing (NGS), have created a paradigm shift in genomics, offering rapid, HTS. Targeted gene panels for specific pathways or skeletal dysplasias are therefore the current gold standard and offer a powerful first‐line diagnostic tool.^(^ ^13^ ^)^ A broader approach can then be undertaken in the form of whole‐exome sequencing (WES) or whole‐genome sequencing (WGS) on the affected individual(s) or as a trio‐sequencing approach, if DNA from parents is available (reviewed elsewhere).^(^ ^4^ ^)^ If multiplex families are available, linkage analysis, alone or coupled with WES/WGS and co‐segregation analysis, can determine the genomic region harboring the causal gene(s)—an approach that has been successfully applied in several HBM disorders.^(^ ^14^, ^15^, ^16^ ^)^ Nevertheless, the success of genetic studies has not been without constraints, due to the lack of large multiplex families, genetic and phenotypic heterogeneity, imprinting, incomplete penetrance, epistasis, and environment interactions. Gene‐burden testing overcomes some of these limitations by comparing the cumulative effects of multiple rare, protein‐altering variants between cases and controls.^(^ ^17^ ^)^ Large‐scale public sequencing databases (eg, Genome Aggregation Database [gnomAD])^(^ ^18^ ^)^ have further supported this notion by providing control sequencing data.

**Fig. 1:**
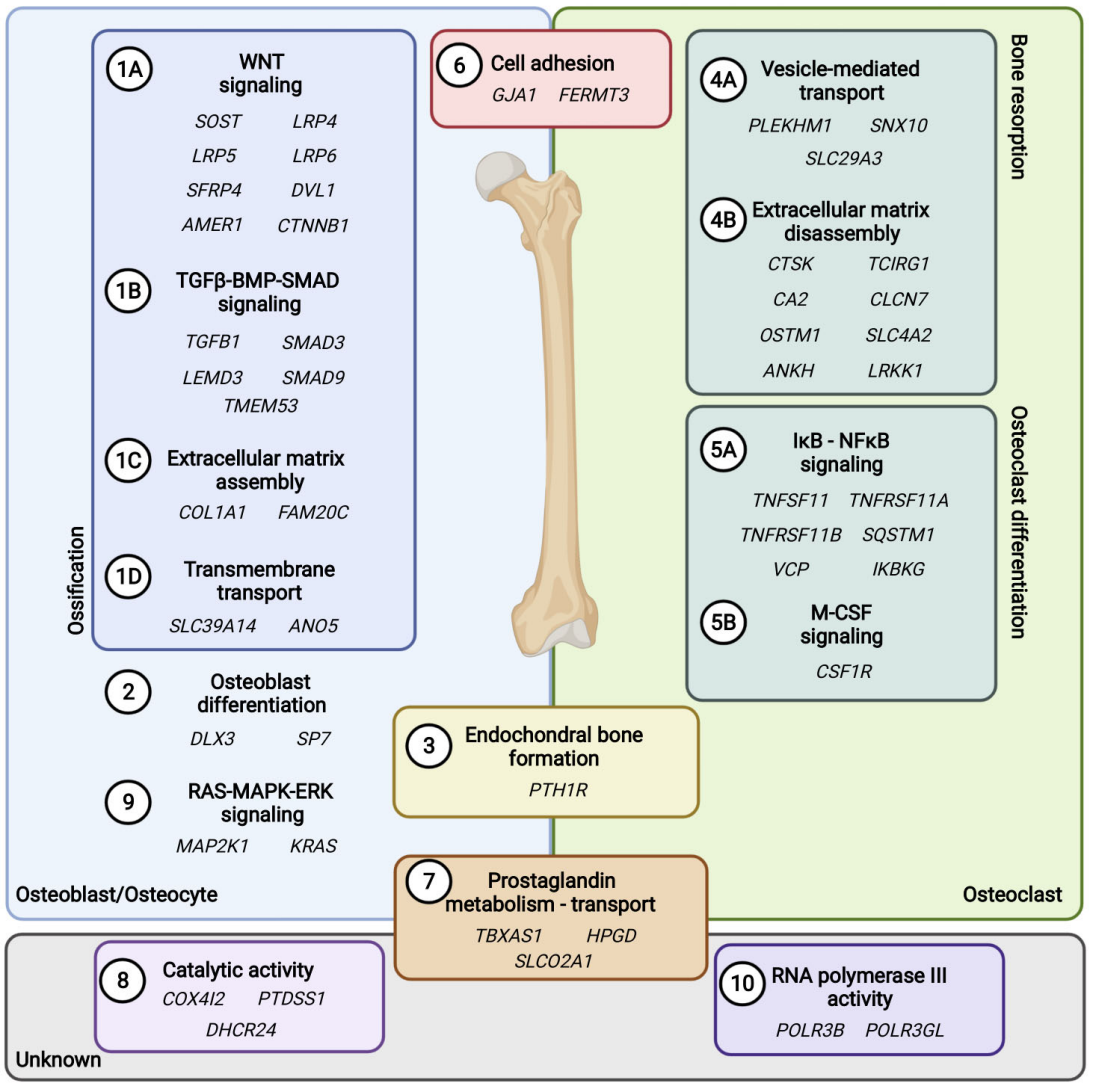
Overview of HBM genes and their associated biological processes and pathways. Graphical overview of the currently known genes that harbor pathogenic variants causing HBM. The genes were allocated to their role in the main bone cell types (or lack of), and subsequently subdivided to a biological process and/or signaling pathway, resulting in 10 groups of HBM genes (numbered). HBM = high bone mass.

Despite these challenges, current gene discovery strategies have so far identified 50 genes as causal for monogenic disorders with significant HBM (Fig. 1). These genes all encode proteins that regulate signaling pathways or biological processes with the potential to increase BMD. Undoubtedly, understanding the etiology of these disorders will inform biological function relevant to bone biology.

### Key biological processes shaped by the study of monogenic HBM disorders

#### WNT/β‐catenin signaling

Genetic knowledge of HBM has shown us the importance of signaling pathways in bone development and homeostasis. A textbook example is the discovery of enhanced canonical WNT/β‐catenin signaling induced by pathogenic variants in *SOST*, *LRP4*, *LRP5*, and *LRP6* in individuals with extreme HBM disorders; ie, sclerosteosis (OMIM 269500; 614305), van Buchem disease (OMIM 239100), craniodiaphyseal dysplasia (OMIM 122860), endosteal hyperostosis (OMIM 144750) and generalized osteosclerosis (OMIM n.a. [not available]) (Fig. 1).^(^ ^6^, ^8^, ^16^, ^19^, ^20^, ^21^ ^)^ These phenotypes revealed a osteoanabolic potential, as this elevated signaling activity resulted in increased bone formation and extremely dense and fracture‐resistant bones.^(^ ^22^ ^)^ In the WNT/β‐catenin pathway, cytoplasmic β‐catenin is phosphorylated by the destruction complex (ie, Axin, GSK‐3β, Disheveled, etc.) which leads to proteasomal degradation, preventing β‐catenin to translocate into the nucleus to regulate gene expression. Activation of WNT/β‐catenin signaling inhibits β‐catenin destruction, enabling translocation into the nucleus and expression of WNT/β‐catenin target genes. HBM disorders affecting WNT/β‐catenin signaling demonstrated that pathogenic variants in these HBM genes mostly result in an intense enhanced osteoblastic response. This may occur not only from pathogenic variants affecting receptor and ligand interactions, but also from variants coding for downstream intracellular components, with HBM also reported in individuals harboring damaging variants in *CTTNB1* (encoding β‐catenin), *AMER1* (*WTX*), and *DVL1* (encoding Disheveled) that can disrupt the cytoplasmic destruction of β‐catenin.^(^ ^23^, ^24^, ^25^ ^)^ In contrast, LoF pathogenic variants in *SFRP4*, encoding the WNT‐sequestering protein sFRP4, were identified in Pyle's disease (OMIM 265900), which is characterized by cortical thinning but increased trabecular bone mass.^(^ ^26^ ^)^ These variants in *SFRP4* led to enhanced signaling in both the canonical and noncanonical arms of the pathway.

#### TGF‐β/BMP‐SMAD signaling

HBM may also result from induced ossification, acting through components of the transforming growth factor β (TGF‐β) and bone morphogenetic protein (BMP); these pathways are highly interlinked by regulating phosphorylation of cytoplasmic SMAD transcription factors (henceforth called the TGF‐β/BMP‐SMAD pathway) (Fig. 1). Pathogenic gain‐of‐function (GoF) variants in *TGFB1* or LoF variants in *LEMD3* and *SMAD9* activate the pathway and generally increase BMD. Moreover, somatic or acquired pathogenic variants affecting TGF‐β/BMP‐SMAD signaling, ie, occurring during early developmental stages or in adult life, can be related to a HBM disorder characterized by a focal rather than generalized increase in ossification. For example, somatic GoF variants in *SMAD3* result in focal pathognomonic lesions of increased bone mass in the endosteal form of melorheostosis.^(^ ^27^ ^)^ Sometimes these clinical aspects of melorheostosis are also detected in osteopoikilosis and dermatoosteopoikilosis (Buschke‐Ollendorff syndrome; OMIM 166700), which are *LEMD3*‐associated HBM disorders.^(^ ^28^ ^)^ Typically, however, melorheostosis is caused by activating somatic variants in members of the rat sarcoma (RAS)‐mitogen‐activated protein kinase (MAPK)‐extracellular signal–regulated kinase (ERK) pathway (*MAP2K1*, *KRAS)*, leading to enhanced osteoblast proliferation.^(^ ^29^, ^30^ ^)^ These findings illustrate that pathways linked to basic cellular processes and which become dysregulated in, eg, oncogenesis, can also cause (mosaic forms of) HBM disorders.

#### Osteoblast differentiation

Besides osteoblast activity, pathogenic variants in genes encoding transcription factors that regulate osteoblast differentiation have also been identified as HBM genes. Pathogenic variants in *DLX3* and *SP7* (encoding Osterix) cause the HBM disorders tricho‐dento‐osseous dysplasia (OMIM 190320) and cranial hyperostosis with long bone fragility (OMIM n.a.), respectively (Fig. 1).^(^ ^31^, ^32^ ^)^ Because transcription factor activity is a multifaceted process, mutations in their corresponding genes can give a wide variety of phenotypes depending on their residual, hypomorphic, or neomorphic activities.

#### Bone resorption

Defects in bone resorption, from altered osteoclast recruitment, differentiation, or resorptive capacity, lead to osteopetrosis, manifest by thicker and/or more dense bones but with greater fragility predisposing to fracture.^(^ ^33^ ^)^ A key role for the nuclear factor kappa‐light‐chain‐enhancer of activated B cells (NF‐κB) signaling in osteoclast differentiation has been clearly illustrated by the osteoclast‐poor forms of osteopetrosis, such as those caused by pathogenic variants in *TNFSF11* (RANKL; OMIM 259710),^(^ ^34^ ^)^ *TNFRSF11A* (RANK; OMIM 612301, OMIM 224300),^(^ ^35^ ^)^ or *IKBKG* (NEMO; OMIM 300291).^(^ ^36^ ^)^ In contrast, osteoclast‐rich forms of osteopetrosis may result from LoF variants in a large group of genes that affect osteoclast function by regulating bone matrix resorption (Fig. 1). For example, impaired function of the proteins encoded by *CAII*, *TCIRG1*, *CLCN7*, and *OSTM1* result in impaired acidification of the mineralized extracellular matrix (ECM).^(^ ^37^, ^38^, ^39^, ^40^, ^41^ ^)^ Other pathogenic variants disturb protein‐trafficking within the osteoclast, altering its ability to perform its resorptive function. These HBM forms include *PLEKHM1*‐related (OMIM 611497; OMIM 618107)^(^ ^42^, ^43^ ^)^ and *SNX10*‐related osteopetrosis (OMIM 615085)^(^ ^44^ ^)^ and dysosteosclerosis caused by *SLC29A3* mutations (OMIM 224300)^(^ ^45^ ^)^ (Fig. 1).

These findings demonstrate that these pathways and processes are not only critical intersections in bone biology but also serve as mutational hotspots for HBM disorders. However, only a few genes have been thoroughly studied. Many of the genes that are poorly understood tend to be linked to (ultra)rare HBM conditions, which together will provide an attractive resource to discover new disease mechanisms.

### Novel biological processes with anabolic potential for bone tissue

During the past decade, rapid progress in genetic screening technologies has enabled the identification of a larger variety of genes and biological processes linked to HBM. For example, pathogenic variants in genes encoding transmembrane transporters can cause HBM diseases but without necessarily causing extraskeletal manifestations. Damaging variants in *SLC39A14* and *ANO5*, both encoding transporters with a prominent function in osteoblasts, are responsible for HBM conditions hyperostosis cranialis interna (OMIM 144755)^(^ ^14^ ^)^ and gnathodiaphyseal dysplasia (OMIM 166260), respectively.^(^ ^46^ ^)^ Similarly for osteoclasts, mutations in *SLC29A3* and *SLC4A2* encoding respective nucleoside and anion transporters cause dysosteosclerosis (OMIM 224300)^(^ ^45^ ^)^ and recessive osteopetrosis, Ikegawa type (OMIM n.a.) (Table S1).^(^ ^47^ ^)^

Interestingly, some HBM genes exert a significant role in the regulation of enzymatic activity, including the enzyme‐encoding genes *COX4I2*, *PTDSS1*, and *DHCR24* associated with exocrine pancreatic insufficiency, dyserythropoietic anemia, and calvarial hyperostosis (OMIM 612714), Lenz‐Majewski hyperostotic dysplasia (OMIM 151050), and desmosterolosis (OMIM 602398), respectively.^(^ ^48^, ^49^, ^50^ ^)^ Pathogenic variants in *HPGD* and *SLCO2A1*, encoding proteins involved in prostaglandin‐related processes, are responsible for a recessive and dominant form of primary hypertrophic osteoarthropathy (OMIM 259100; 161700), respectively.^(^ ^51^ ^)^ This illustrates that HBM genes belonging to the same group, and hence encoding proteins that regulate a similar biological process can result in similar phenotypes. Similarly, *POLR3B* and *POLR3GL* both encode for subunits of the DNA‐directed RNA Polymerase III enzyme, and pathogenic variants in both genes cause HBM diseases characterized by endosteal hyperostosis (OMIM 614381; 619234).^(^ ^52^ ^)^ Overall, these more unexpected biological processes harbor novel potential to increase bone mass.

### Classification of HBM disorders according to their perturbed biological processes

As alluded in the previous section, HBM genes can be clustered based on shared biological functions (Fig. 1). For this review, we classified the 50 known HBM genes and their 59 associated disorders according to their established role in a signaling pathway and/or biological process (Table 1; Table S1). We used uniform Gene Ontology (GO) accession numbers (http://geneontology.org/) to create 10 distinct HBM (sub)groups. Moreover, GO identifiers were kept as broad as possible so that new genes can be added to existing HBM (sub)groups in the future (Table 1).

**Table 1:** Classification of HBM Genes and Their Associated Disorders According to Biological Process and/or Pathway

Biological process (GO accession number)/gene	Disorder	Inheritance	OMIM	Nosology group
1. Regulation of ossification (GO:0030278) group				
1A. Regulation of Wnt signaling (GO:0008590) subgroup				
*SOST*	Sclerosteosis, type 1	AR	269500	24
	van Buchem disease	AR	239100	24
	Craniodiaphyseal dysplasia	AD	122860	24
*LRP4*	Sclerosteosis, type 2	AR, AD	614305	24
*LRP5*	Endosteal hyperostosis/Osteosclerosis	AD	144750	24
*LRP6*	Generalized osteosclerosis	AD	n.a.	n.a.
*SFRP4*	Metaphyseal dysplasia (Pyle's disease)	AR	265900	24
*DVL1*	Robinow syndrome, with osteosclerosis	AD	616331	17
*AMER1*	Osteopathia striata with cranial sclerosis	XLD	300373	24
*CTNNB1*	Osteosclerosis and adrenocortical neoplasia	AD/mosaic	n.a.	n.a.
1B. Regulation of TGF‐β‐BMP‐SMAD signaling (GO:0017015) subgroup				
*TGFB1*	Diaphyseal dysplasia (Camurati‐Engelmann disease)	AD	131300	24
*LEMD3*	Osteopoikilosis, with or without melorheostosis	AD	166700	24
	Buschke‐Ollendorff syndrome (dermatoosteopoikilosis), with or without melorheostosis	AD	166700	24
*SMAD3*	Melorheostosis, endosteal	n.a	n.a.	n.a.
*SMAD9*	Generalized osteosclerosis	AD	n.a.	n.a.
*TMEM53*	Craniotubular dysplasia, Ikegawa type	AR	619727	n.a.
1C. Regulation of extracellular matrix assembly (GO:1901201) subgroup				
*COL1A1*	Infantile cortical hyperostosis (Caffey disease)	AD	114000	22
*FAM20C*	Osteosclerotic bone dysplasia, lethal (Raine syndrome)	AR	259775	22
1D. Regulation of transmembrane transport (GO:0034762) subgroup				
*SLC39A14*	Hyperostosis cranialis interna	AD	144755	n.a.
*ANO5*	Gnathodiaphyseal dysplasia	AD	166260	25
2. Regulation of osteoblast differentiation (GO:0045667) group				
*DLX3*	Tricho‐dento‐osseous syndrome	AD	190320	24
*SP7*	Cranial hyperostosis and long bone fragility	AD, dNO	n.a.	n.a.
3. Regulation of endochondral ossification (GO:0001958) group				
*PTH1R*	Blomstrand chondrodysplasia	AR	215045	22
4. Regulation of bone resorption (GO:0045124) group				
4A. Regulation of vesicle‐mediated transport (GO:0060627) subgroup				
*PLEKHM1*	Osteopetrosis, type OPTB6	AR	611497	23
	Osteopetrosis, type OPTA3	AD	618107	23
*SNX10*	Osteopetrosis, type OPTB8	AR	615085	23
*SLC29A3*	Dysosteosclerosis	AR	224300	23
4B. Regulation of extracellular matrix disassembly (GO:0010715) subgroup				
*CTSK*	Pycnodysostosis	AR	265800	23
*TCIRG1*	Osteopetrosis, type OPTB1	AR	259700	23
*CA2*	Osteopetrosis, type OPTB3	AR	259730	23
*CLCN7*	Osteopetrosis, type OPTB4	AR	611490	23
	Osteopetrosis, type OPTA2	AD	166600	23
*OSTM1*	Osteopetrosis, type OPTB5	AR	259720	23
*SLC4A2*	Osteopetrosis, Ikegawa type	AR	n.a	n.a.
*ANKH*	Craniometaphyseal dysplasia	AD	123000	24
*LRRK1*	Osteosclerotic metaphyseal dysplasia	AR	615198	23
5. Regulation of osteoclast differentiation (GO:0045670) group				
5A. Regulation of I‐κB kinase/NF‐κB signaling (GO:0043122) subgroup				
*TNFSF11*	Osteopetrosis, type OPTB2	AR	259710	23
*TNFRSF11A*	Osteopetrosis, type OPTB7	AR	612301	23
	Dysosteosclerosis	AR	224300	23
*TNFRSF11B*	Juvenile Paget's disease	AR	239000	24
*SQSTM1*	Paget's disease of bone	AD	167250	n.a.
*VCP*	Inclusion body myopathy with early‐onset Paget disease and frontotemporal dementia 1	AD	167320	n.a.
*IKBKG*	Osteopetrosis, with lymphedema, ectodermal dysplasia, anhidrotic, and immunodeficiency (OLEDAID)	XLR	300291	23
5B. Regulation of macrophage colony‐stimulating factor signaling pathway (GO:1902226) subgroup				
*CSF1R*	Dysosteosclerosis, brain abnormalities, neurodegeneration	AR	618476	23
6. Regulation of cell adhesion (GO:0030155) group				
*GJA1*	Craniometaphyseal dysplasia	AR	218400	24
	Oculodentoosseous dysplasia, mild type	AD	164200	24
	Oculodentoosseous dysplasia, severe type	AR	257850	24
*FERMT3*	Osteopetrosis with defective leukocyte adhesion	AR	612840	23
7. Regulation of prostaglandin metabolism or transport (GO:0001516; GO:0015732) group				
*TBXAS1*	Ghosal hematodiaphyseal dysplasia	AR	231095	24
*HPGD*	Primary hypertrophic osteoarthropathy	AR	259100	24
*SLCO2A1*	Primary hypertrophic osteoarthropathy	AD	167100	24
8. Regulation of catalytic activity (GO:0050790) group				
*COX4I2*	Calvarial hyperostosis, with pancreatic insufficiency and dyserythropoietic anemia	AR	612714	n.a.
*PTDSS1*	Lenz‐Majewski hyperostotic dysplasia	AD	151050	24
*DHCR24*	Desmosterolosis	AR	602398	22
9. Regulation of RAS‐MAPK–ERK signaling (GO:0046578; GO:0043408) group				
*MAP2K1*	Melorheostosis, isolated, somatic mosaic	n.a.	155950	24
*KRAS*	Melorheostosis, isolated, somatic mosaic	n.a.	n.a.	n.a.
10. Regulation of RNA Polymerase III activity (GO:1903622) group				
*POLR3B*	Cerebellar hypoplasia with endosteal hyperostosis	AR	614381	24
*POLR3GL*	Short stature, oligodontia, dysmorphic facies, and motor delay with endosteal sclerosis	AR	619234	n.a.

AD = autosomal dominant; AR = autosomal recessive; DN = dominant negative; dNO = de novo; GO = Gene Ontology; HDM = high bone mass; n.a. = not available; OMIM = Online Mendelian Inheritance in Man; XLD = X‐linked dominant; XLR = X‐linked recessive.

Some HBM groups are very evident: “Positive regulation of ossification” (GO:0045778, HBM group 1), containing key pathways such as “Regulation of Wnt signaling” (GO:0008590, subgroup 1A) and “Regulation of TGF‐β‐BMP‐SMAD signaling” (GO:0017015, subgroup 1B). Similarly, genes involved in the “Regulation of bone resorption” were also grouped (GO:0045779, HBM group 4). Smaller HBM groups so far contain the poorly understood HBM genes (eg, *COX4I2*, *GJA1*, *FERMT3*, *PTDSS1*) involved in processes such as “Regulation of cell adhesion” (GO:0030155, HBM group 6) and “Regulation of enzymatic catalytic activity” (GO:0050790, HBM group 8).

We believe that this classification based on biological function (Table 1) can complement the existing and more clinically‐based classification of all genetic skeletal disorders by the International Skeletal Dysplasia Society (ISDS) and may help in determining the genetic background and subsequent clinical approach for certain HBM phenotypes.^(^ ^53^ ^)^ Identification of new HBM genes within the known subgroups could help in further functional characterization or may create new subgroups when novel biological processes are associated with HBM.

## Understanding HBM Mechanisms through Functional Genomics

**Fig. 2:**
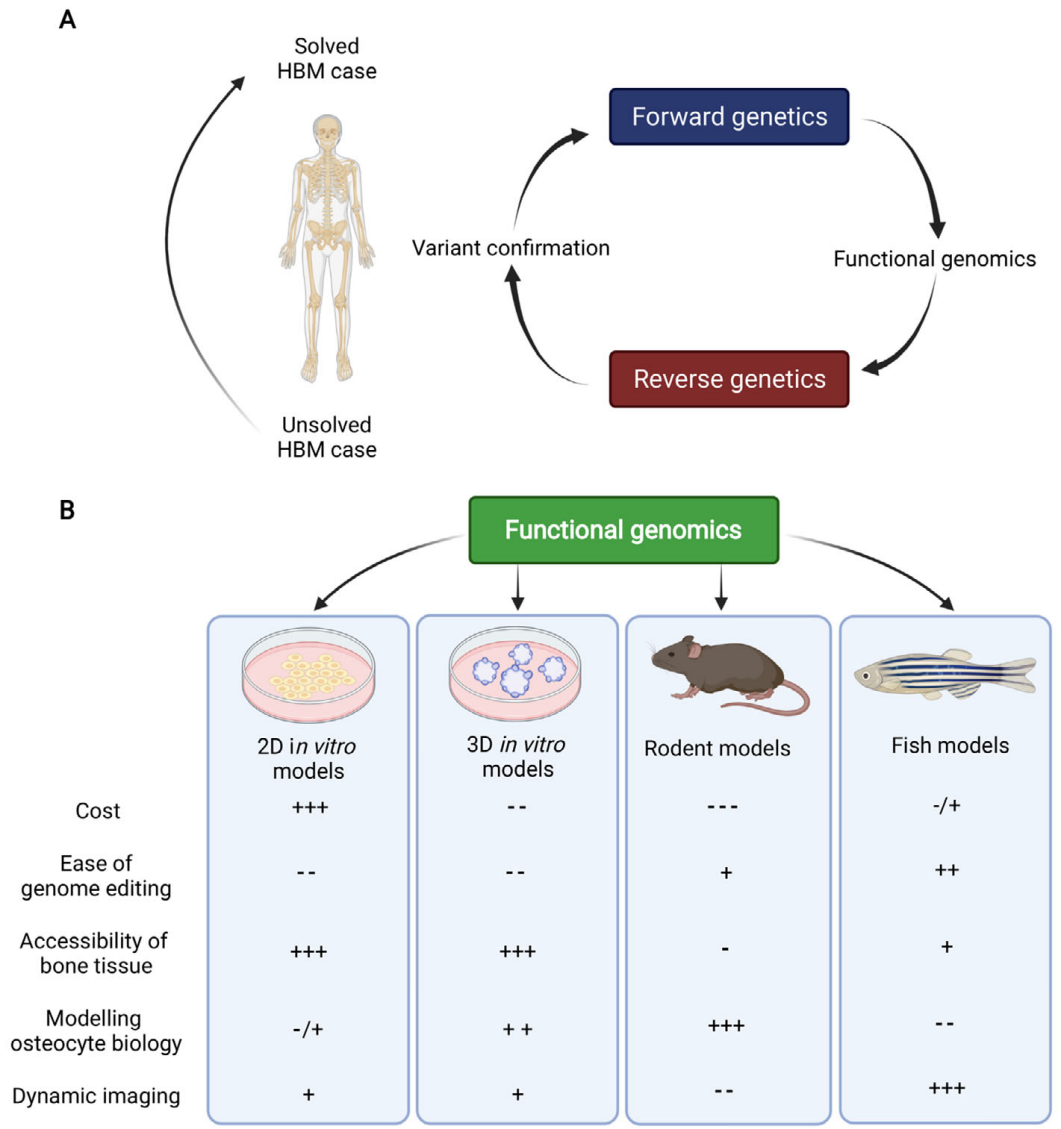
Overview of forward versus reverse genetics and functional genomics tools for HBM research. (*A*) the forward and reverse genetic research cycle to discover new genes with HBM causing variants allowing to solve genetically unexplained HBM cases in the clinic. (*B*) The functional genomic toolbox at the disposal of basic and translational health scientists encompassing, but not limited to 2D and 3D in vitro models, mouse and rats, and zebrafish. The + stands for more advantageous and − for more disadvantageous relative to the other common model systems used in the field. HBM = high bone mass.

Forward genetic approaches (from phenotype to genotype) have been the main driver of our molecular and functional understanding of HBM disorders. Substantial technological developments now allow larger‐scale testing of molecular pathways on a systems level; ie, through functional genomics. This means that a “reverse genetic” approach is now feasible, where a genotype is used to understand the molecular and metabolic makeup of skeletal phenotypes (Fig. 2A). By deploying such an approach, one can reveal molecular, regulatory, and genetic networks and mechanisms that are dysregulated due to the genetic defect causing HBM.

### Omic technologies as a basis in functional genomics

In the era of omics, the wide array of available in vitro and in vivo model systems provide functional genomics tools to scrutinize HBM disease pathways. Omics allow capturing the molecular architecture of a cell or a tissue in its entirety in a “hypothesis‐free” setting. Those in‐depth profiles of a “biological activity” (eg, via transcriptomics [RNA expression], proteomics [protein abundance], or metabolomics [enzymatic activity of proteins]) can be linked to available genomic and epigenomic datasets that perhaps could be described as “functional potential” data. The combined output can then show that certain “functional predictions” (ie, genetic variants, and/or histone methylation) are indeed regulating a biological activity involving HBM pathophysiology.^(^ ^54^, ^55^ ^)^

A few important notes should be considered regarding the complex tissue of bone: (i) bone contains many different cell types; (ii) it is relatively time‐consuming and difficult to acquire bone tissue from affected cases/controls, or from in vivo models; (iii) bone has major two forms of formation (intramembranous or endochondral ossification); and (iv) each bone element has a unique location/microenvironment in the skeleton which may be subject to its own unique gene expression and protein composition signature. These practical issues provide a (partial) explanation why there have been relatively few bone omic studies involving HBM published in the past few years (Table 2).

**Table 2:** Overview of Omic Studies and Investigated Biological Processes That Can Model Characteristics of Bone Anabolism in the Main Model Systems

Omic	Method	Species	Tissue or cell type	Bulk or Sc	Genotype and/or conditions	Process	Highlighted pathways, regulatory nodes, and/or group of factors	Citation
T	Microarray	Human	Mcy	Bulk	H/L‐BMD	Oc differentiation	RIG‐I like receptor, fatty acid metabolism	(56)
T	Microarray	Human	MSC, Ob	Bulk	Co‐culture	Ob differentiation	Collagen synthesis, BMP pathway	(57)
T	RNAseq	Human	Ob	Sc	osteoarthritis and osteopenia	Gene expression during disease	NR4A2/1, COL1A1, SPARC, RUNX2, BGALP, VCAM1, LEPR	(58)
T	RNAseq	Mouse	BM Adpc	Bulk	*Ddr2* ^*fl/fl*^ *;Adipoq‐Cre*	GPCR signaling	Adcy5‐cAMP‐PKA signaling	(59)
T	RNAseq	Mouse	Ob	Bulk	*Lrp5* ^*fl/fl*^ *;Lrp6* ^*fl/fl*^ *;UBC‐Cre‐ER* ^*T2*^	Wnt3a and LRP5/6 signaling	WNT signaling, TGF‐β signaling, MAPK signaling, ECM organization, focal adhesion	(60)
T	Microarray	Mouse	Mcy, Oc	Bulk	*Nfatc1* ^*fl/fl*^ *;Msx1‐Cre*	Oc differentiation	Calcineurin, Rankl, bone resorption	(61)
T	RNAseq	Mouse	Ob	Sc	*R26R‐Lyn‐Venus;Col1a1‐Cre*	Ob differentiation	Cdc34, Cxcl12, Dlx5, Sost, Sp7	(62)
T	RNAseq	Mouse	Adpc	Bulk	*iDTR* ^*fl/fl*^ *;Adipoq‐Cre*	Dynamics between Adpc and Ob	BMP signaling, IGF signaling, ECM synthesis	(63)
T	RNAseq	Mouse	Ob	Bulk	*Cdc73* ^*fl/fl*^ *;Ocn‐Cre*	Bone remodeling	MAPK signaling, collagen processing	(64)
T	Microarray	Mouse	Ob	Bulk	*Lrp5* ^*fl/fl*^ *;Ocn‐Cre*	Fatty acid metabolism	Ob differentiation, fatty acid synthesis	(65)
T	Microarray	Mouse	Ocy454	Bulk	WT	High vs. low *Sost* expressing sub‐clones	Carbonic anhydrase, oxidative stress	(66)
T	Microarray	Mouse	Ocy	Bulk	*ERα* ^*fl/fl*^ *;Dmp1‐Cre*	ERα signaling	Secreted (glyco)proteins, ECM, sost1dc	(67)
T	Microarray	Mouse	Cortical WBE	Bulk	*Phex* ^*−/−*^	Fgf23 production and mineralization	CA pathway, ECM synthesis, BMP signaling, IGF signaling, cell adhesion	(68)
T	RNAseq	Mouse	Skull WBEs	Bulk	*Twist1* ^+/−^	Osteogenesis	Fgf23, bone mineralization	(69)
T	RNAseq	Mouse	SPC	Sc	WT, Rosiglitazone, irradiation, fracture	SPC differentiation: Ob and Adpc dynamics	Notch signaling, Cathepsin K, Twist1, Atf4, Klf4, Hoxb2, Npdc1, Mef2c	(70)
T	RNAseq	Mouse	Endochondral WBE	Sc	WT, fracture healing	MSC derived Septoclasts	Proteoglycans, MMP, Notch signaling, cell‐matrix interactions	(71)
T	Microarray	Mouse	Endochondral WBE	Bulk	*p27* ^*−/−*^	Ob differentiation	Sonic Hedgehog‐Gli‐Bmi1 signaling, p130‐E2F4	(72)
T	RNAseq	Mouse	WBE, Oc, VT	Bulk	*Clnc7* ^*G213R*^	Osteopetrosis, type OPTA2	JAK–STAT signaling, cytokine, hematopoiesis	(73)
T	RNAseq, GSA	Mouse & Human	Ocy	Bulk	WT	Bone homeostasis	WNT signaling, BMP signaling, ECM organization, angiogenesis, axon development	(74)
T	RNAseq	Mouse, rat, macaque	Ocy	Bulk	WT	Cross‐species regulation of bone homeostasis	Regulation of bone remodeling and BMD	(75)
T	Microarray	Rat	Calvaria WBE	Bulk	WT	Bone healing	Focal adhesion, ECM‐receptor interaction, TNF signaling, Hippo signaling	(76)
T	Microarray	Zebrafish	CF	Bulk	WT	Ob differentiation	ECM synthesis, WNT signaling, SMAD‐BMP signaling	(77)
T	RNAseq	Medaka	Ob, CF	Bulk	*rankl:HSE:CFP*	Oc and Ob differentiation	ECM degradation, MMP, ECM‐receptor interactions, cell cycle	(78)
T	RNAseq	Zebrafish	CNCC	Sc	WT	Craniofacial development	WNT signaling, FOXD, v‐ATPases	(79)
T, Met, Mu	Microarray, meDIPseq	Human	BMSC	Bulk	H/L‐BMD	BMSC differentiation	MicroRNAs, AKT‐STAT signaling, FAM50A, ZNF473, TMEM55B, FLT3	(80)
T, E, Met	RNAseq, WGmetseq	Human	iPSCs	Bulk	*CLCN7* ^*R286W*^	Osteopetrosis, type OPTA2, transcriptional programming	TNF signaling, Ras signaling, FOXO	(81)
T, G	RNAseq, GWAS	Mouse, Human	BMSC	Sc	*Cxcl12‐eGFP* and *Rspo3* ^*fl/fl*^ *;Runx2Cre*	BMSC differentiation	Proteasomal degradation of WNT receptors	(82)
T, G	RNAseq, GWAS	Mouse, Human	Cortical WBE	Bulk	WT	Aging	PI3K‐AKT signaling, focal adhesion, cell adhesion, ECM synthesis, WNT signaling, TGF‐β signaling	(83)
T, G	RNAseq, GSA	Zebrafish Human	ES	Bulk	WT	Ob differentiation	Collagen processing, ECM synthesis, focal adhesion, hedgehog signaling, IGF signaling	(84)
T, E	RNAseq, ATACseq	Zebrafish	CF	Bulk	WT	Ob differentiation	Cell cycle process, ECM organization, cholesterol biosynthesis	(85)
T, E	RNAseq, snATACseq	Zebrafish	CNCC	Sc	WT	CNCC differentiation during lifespan	ECM organization, BMP signaling, WNT signaling, NFAT, RUNX, CXCL12	(86)
T, P, Mb	RNAseq, LC–MS/MS	Zebrafish	CF	Bulk	WT	Ob differentiation	Retinoic acid, WNT signaling, FGF signaling	(87)
P	LC–MS/MS	Human	Ob, BMSC	Bulk	Dexamethasone and hyaluronic acid	Ob‐released matrix vesicles	ECM synthesis, Integrin, PPARɣ, CXCR4, MAPK–ERK signaling, EIF2	(88)
P	LC–MS/MS	Zebrafish	CF	Bulk	Prednisolone	Ob differentiation	ECM synthesis, focal adhesion, ion binding, secretory pathway	(89)
P	MS	Zebrafish	WBE, CF	Bulk	WT	Bone maturation and aging	ECM synthesis, WNT signaling	(90)
P	LC–MS/MS, MALDI‐MS	Zebrafish	CF	Bulk	WT	Ob differentiation	Focal adhesion, regulation of Actin cytoskeleton	(91)
P	MALDI‐MS	Zebrafish	CF	Bulk	WT	Ob differentiation	Focal adhesion, immune response, cytoskeleton	(92)
G	GWAS	Mouse	Som	WG	WT	Aging	Osteoblast differentiation, BMP signaling	(93)
Mb	NMR	Human	Serum	Bulk	Unexplained HBM	Bone turnover markers	β‐C‐terminal telopeptide of type‐I collagen, citric acid	(94)

Adpc = adipocyte; ATACseq = assay for transposase accessible chromatin sequencing; BMSC = bone marrow stem cell; (H/L)BMD = (high/low) bone mineral density; CA = carbonic anhydrase; CF = caudal fin; CNNC = cranial neural crest cell; DTR = diphtheria toxin receptor; E = epigenomic; ECM = extracellular matrix; ERα = estrogen‐receptor α; *fl/fl*: *flox/flox*; G = genomic; GPCR = G‐protein coupled receptor; GSA = gene set analysis; GWAS = genomewide association study; IGF = insulin‐like growth factor; LC–MS = liquid chromatography–mass spectrometry; MALDI = matrix‐assisted laser desorption/ionization; Mb = metabolomic; Mcy = monocyte; Met = methylomics; MS = mass spectrometry; MSC = mesenchymal stem cell; Mu = MicroRNAomic; NMR = proton nuclear magnetic resonance spectroscopy; Ob = osteoblast; Oc = osteoclast; Ocy = osteocyte; P = proteomic; RNAseq = RNA‐sequencing; Sc = single‐cell; Sn = single‐nucleus; Som = somatic; SPC = skeletal progenitor cell; T = transcriptomic; VT = visceral tissue; WBE = whole‐bone element; WG = whole‐genome; WGmetseq = whole‐genome methylome sequencing; WT = wild‐type.

The overarching strength of omics is that they widely capture “biological activity” and create molecular systems or signatures that reflect certain disease states. Transcriptome technologies, such as microarray hybridization technology and RNA‐sequencing (RNAseq) are used most frequently in the HBM field (Table 2). In recent years, RNAseq of isolated tissue (bulk RNAseq) or single cells isolated from a tissue (scRNAseq) have been more widely deployed and allow to capture the spatiotemporal expression profile or a comparison of control versus disease/treatment. scRNAseq especially generates complex profiles that define distinct cell populations in an unbiased way. This allows exploration of mechanisms caused by minority cell populations or by changes in the proportion of bone lineages, which can be hidden in a bulk strategy. These transcriptional signatures of cell populations can therefore reveal the heterogeneity,^(^ ^95^ ^)^ even after fluorescence‐activated cell sorting (FACS).

Although transcriptomic studies are one strategy to explore pathological changes in bone cells or tissue, other mechanisms may be better studied by proteomic, epigenomic, and/or metabolomic approaches; eg, processes that involve cellular stress, transcription factor binding, or environmentally induced HBM after exposure to excessive levels of sodium fluoride (skeletal fluorosis).^(^ ^96^ ^)^ These less common omic strategies are yet to be conducted widely in bone, but they have great potential.

The available model systems and methods of in‐depth phenotyping to study bone mass have been extensively reviewed previously by the GEMSTONE working groups and others.^(^ ^3^, ^4^, ^97^, ^98^, ^99^ ^)^ Here, we primarily focus on the state‐of‐the‐art in key lab‐based model systems and the potential of combining multiple omic assessments in multiple model systems for the HBM field.

### State‐of‐the‐art functional genomics approaches

#### 2D in vitro cultures

2D monocultures and co‐cultures of bone cell types are a common means of generating functional data rapidly to understand various genetic consequences (Fig. 2B). Such cultures allow readouts of, eg, cell metabolism, ECM formation, and subcellular localization of proteins, which is difficult to capture in vivo.^(^ ^100^, ^101^, ^102^, ^103^, ^104^, ^105^ ^)^ For this purpose, various cell lines for all bone cell types have been created and have been extensively reviewed.^(^ ^106^, ^107^, ^108^, ^109^ ^)^ As an example pertinent to the study of HBM, the Ocy454 cell line is a *Dmp*‐positive (*Dmp* ^+^) osteocytic cell line that expresses elevated levels of *Sost*, making it a model to study the effects of mechanical loading.^(^ ^105^ ^)^

Transcriptome microarray profiling revealed *CA3* (encoding carbonic anhydrase III) as a novel marker of differentiated osteocytes in high *Sost*‐expressing clones, next to typical markers such as *Dmp1* and *Phex*. This led to the understanding that CAIII protects osteocytes from oxidative stress.^(^ ^66^ ^)^ Interestingly, expression studies also demonstrated that sclerostin induces *CA2* (encoding carbonic anhydrase II) to regulate bone mineral release in MLO‐Y4 cells, another osteocytic cell line.^(^ ^100^ ^)^ This shows that genes coding for enzymes, like carbonic anhydrases, can unexpectedly be important for cells from the mesenchymal lineage. One good example is *CA2*, traditionally classified as an osteoclast gene harboring mutations causal for a severe form of osteopetrosis (OMIM 259730).

#### Rodent models

Mouse and rat models have been widely used as an in vivo model for the human skeletal system. They possess all the relevant skeletal cell types, types of bone, and genes between humans and rodents have high homology (Fig. 2B).^(^ ^106^ ^)^ Mouse models have delivered great successes in bone research, for example in deciphering the WNT/β‐catenin and NF‐κB pathways, by using cellular and dynamic histomorphometric methods, three‐point bending assays, as described.^(^ ^3^, ^110^, ^111^ ^)^ Here, we report a list of 56 transgenic mouse models for 22 known HBM genes and intriguingly, an additional 80 transgenic mouse models covering 56 genes, in which no pathogenic variants have been identified in humans with a form of HBM so far (Table S2). We also identified 20 studies that used mouse‐derived or rat‐derived bone tissue for omic assessments to model aspects of HBM (Table 2).

Recently, another study using bulk RNAseq characterized an “osteocyte transcriptome signature” (OTS) (Table 2) using sequence data from bone matrix‐embedded cells with high *Sost* expression. Genes that have a highly enriched expression in osteocytes included many associated with skeletal diseases (such as osteogenesis imperfecta and sclerosteosis) and were often associated with common skeletal diseases (such as osteoporosis and osteoarthritis).^(^ ^74^ ^)^ Moreover, the study showed that the OTS dynamically changes during skeletal maturation and is sex dependent. The OTS will provide a powerful resource of reference osteocyte genes for future HBM studies. Bulk RNAseq approaches also allow identifying novel regulatory mechanisms yet not associated with HBM, as is demonstrated with Wnt3a dynamically interacting with the Lrp5 and Lrp6 receptors to alter Wnt signaling pathway activation.^(^ ^60^ ^)^

In mice, an scRNAseq approach on FACS *Col1a1*‐expressing (*Col1a1* ^+^) cells explored the concept of osteoblast heterogeneity. Functional annotation resulted in the identification of four clusters; ie, clusters 1–3 captured active bone‐forming osteoblasts in different maturational stages whereas cluster 4 captured fewer active osteoblasts with progenitor properties.^(^ ^62^ ^)^ Biological processes most significantly enriched in these clusters were positive regulation of cell cycle (cluster 1; GO:0045787), endochondral ossification (cluster 2; GO:0001958), chondrocyte differentiation (cluster 3; GO:0002062), and cell adhesion mediated by integrin (cluster 4; GO:0033627).^(^ ^62^ ^)^ A similar strategy was also deployed to understand the role of fracture risk factor *RSPO3* in mesenchymal skeletal stem cell populations fine tuning osteoblastic and adipogenic cell fates.^(^ ^82^ ^)^ Recently, an scRNAseq assessment also identified cartilage and noncalcified bone matrix resorbing cells, called septoclasts, predominantly located at the chondro‐osseous border, which are derived from nonhematopoietic lineages but express *Ctsk* and *Fabp5*.^(^ ^71^ ^)^ Importantly, septoclasts were also involved in fracture repair of endochondral bone. These studies showed that scRNAseq is an extremely valuable tool to find mechanisms and new cell populations that are difficult to capture.

Finally, osteoclasts from the *Clcn7* ^*G213R*^ mouse model with autosomal dominant osteopetrosis (OMIM 166600) have also been analyzed with bulk RNAseq.^(^ ^73^ ^)^ Biological processes enriched in *Clcn7* ^*G213R*^ osteoclasts included response to stimulus (GO:0050896), extracellular matrix organization (GO:0030198) and cell adhesion (GO:0007155), whereas underrepresented processes included RNA processing (GO:0006396), messenger RNA (mRNA) processing (GO:0006397) and cellular response to DNA damage stimulus (GO:0006974). Bulk RNAseq of other tissues affected in osteopetrosis patients (eg, brain, kidney, liver) was also performed to uncover biomarkers for follow‐up of *CLCN7*‐related osteopetrosis patients in future experimental clinical trials.^(^ ^73^ ^)^

### Emerging functional genomics model systems

#### 3D modeling of bone tissue in vitro

One of the holy grails in the bone field is to accurately mimic bone's in vivo complexity in a controlled in vitro laboratory setting. Beyond advancing scientific knowledge per se, this would enable refinement, reduction, and replacement of animals in research (3Rs principle). Although indirect, transwell, and/or direct co‐cultures of osteoblasts, osteocytes, and osteoclasts have been widely used, these approaches can be challenging; eg, they often require complex matrix coatings.^(^ ^112^, ^113^ ^)^ To address this, organoids and three‐dimensional (3D) tissue culture strategies have been proposed. Recently, two exciting organoid systems have been developed with relevance for the HBM field. An organoid of woven bone can track the differentiation process from bone marrow–derived stem cells (BMSCs) to osteocytes in a silk fibroin scaffold‐based 3D setting. New mineralized collagen matrix was visualized with advanced electron microscopy techniques showing remarkable similarities with woven bone in situ.^(^ ^114^ ^)^

Second, an organoid of trabecular bone was derived from mesenchymal stromal cells separated by spacers, in a demineralized bone paper scaffold‐based 3D environment; the spacers then allowed exposure to osteoclasts, thus replicating bone remodeling in vitro.^(^ ^115^ ^)^ As an example in HBM, such in vitro tissue engineering approaches have been used to study osteopetrosis caused by LoF *TNFSF11* (*RANKL*) mutations in *Rankl* ^*−/−*^ mice.^(^ ^116^, ^117^ ^)^ These culture systems are often derived from induced pluripotent stem cells (iPSCs), or from BMSCs harvested from consented patients, with subsequent differentiation into skeletal cell types.^(^ ^118^, ^119^ ^)^ However, iPSCs derived from individuals with genetically unexplained HBM could also be used to gain mechanistic insights into the cellular and molecular causes of their disease. Thus, organoids have immense potential, but are still to be established as a common methodology, at least in part due to expense; currently costing ~US$1000 per culture, though likely to fall with increased use and protocol refinement (Fig. 2).^(^ ^120^, ^121^ ^)^

#### Fish models

Zebrafish (*Danio rerio*), or occasionally medaka fish (*Oryzias latipes*), are also used to model human diseases. They are relatively cheap to house, amenable to genetic and pharmacological manipulation, and accessible for skeletal imaging (Fig. 2B).^(^ ^122^ ^)^ More than 85% of human disease causing genes have orthologues in zebrafish and their skeletal physiology shows strong similarities to mammals.^(^ ^123^ ^)^ Their mineralized endoskeleton also responds to *sost‐*regulated remodeling after loading.^(^ ^124^, ^125^ ^)^ Adult zebrafish also have a mineralized exoskeleton that enables ex vivo tracking of bone regeneration and healing.^(^ ^126^ ^)^ To date, there is a vast library of transgenic reporter and mutant zebrafish available that has been shown to accurately model various skeletal diseases (Zebrafish Information Network [ZFIN]; www.zfin.org), allowing bone cell populations to be imaged, FACS isolated, or manipulated.^(^ ^124^, ^127^, ^128^ ^)^ Zebrafish can also model high BMD^(^ ^84^, ^129^, ^130^, ^131^ ^)^; eg, an osteopetrosis‐like phenotype in *mmp9* ^−/−^;*mmp13b* ^*−/−*^ double mutant fish,^(^ ^78^ ^)^ *CSF1R*‐related dysosteosclerosis (OMIM 618476),^(^ ^132^, ^133^ ^)^ and *PTDSS1*‐related Lenz–Majewski hyperostotic dysplasia (OMIM 151050).^(^ ^134^ ^)^

##### Exploiting the zebrafish lifespan to understand spatiotemporal and molecular causes of HBM

Initial zebrafish development is rapid with the first skeletal progenitor cells in the form of neural crest cells appear around the first day of development. During neural crest cell migration, cranial neural crest cell (CNCC) progenitors form parts of the craniofacial skeleton.^(^ ^132^, ^135^ ^)^ Neurocristopathies are a group of disorders where the migration of neural crest cells is perturbed, which can affect many tissues, including skeletal elements in the face and jaw, teeth, bone marrow (hematopoietic lineage), and ears.^(^ ^136^ ^)^ Additionally, neural crest cells are a multipotent cell population and its migration is also pivotal for proper neurological, pigment, heart, and sensory development.^(^ ^137^, ^138^ ^)^ Some HBM disorders with significant craniofacial involvement have characteristics of neurocristopathies, such as the mandible enlargement seen in van Buchem disease patients. Similarly, Lenz‐Majewski hyperostosis, gnathodiaphyseal dysplasia, Robinow syndrome, and desmosterolosis lie within the neurocristopathy spectrum. *DLX3* is also a well‐known factor in neural crest cells of which mutations result in tricho‐dento‐osseous syndrome (Table 1).^(^ ^139^ ^)^ Because neural crest migration and their derivatives can be visualized both in real time and throughout the zebrafish lifespan, there is a great potential to fundamentally understand the early processes underlying these disorders.

An scRNAseq approach showed transcriptional heterogeneity among CNCCs with distinct cell populations committed to become skeletal progenitors, melanocytes, or neuronal glial cells.^(^ ^79^ ^)^ Another study linked transcriptomic and epigenomic datasets focused on longitudinal specification and diversification potential of single CNCCs fate throughout the zebrafish lifespan.^(^ ^86^ ^)^ With a single‐nuclei assay for transposase accessible chromatin sequencing (snATACseq) and scRNAseq technologies it is possible to match chromatin accessibility (potential for activity) with gene expression (activity) in single cells during cell type differentiation.^(^ ^86^ ^)^ Using omic approaches could provide a fundamental understanding of the dysregulated gene networks during CNCC migration and cell differentiation in zebrafish mutants of HBM with neurocristopathological elements^(^ ^136^ ^)^ or poorly studied multitissue disorders (ie, HBM group 8, Table 1).

SMAD9, encoded by the HBM gene *SMAD9*, is mostly known for as a BMP‐signaling transcriptional inhibitor.^(^ ^140^ ^)^ Studying Smad9 in zebrafish uncovered that smad9 inhibits osteochondral precursor differentiation, which responded to pharmacological treatment.^(^ ^141^ ^)^ Zebrafish skeletons continue growing throughout life, enabling facets of growing bone to be tracked in living fish over time, as demonstrated by the impaired formation of calvarial sutures in *sp7‐*deficient zebrafish.^(^ ^142^, ^143^ ^)^ Proteomics of the acellular ECM of bone from skull, axial, and exoskeletal fin rays from different developmental stages showed that ECM synthesis proteins were abundant at all stages and that endochondral ossification proteins became less abundant with age whereas proteins involving ECM synthesis increased their relative abundance.^(^ ^90^ ^)^ Following the growth and maturation of bone in an adult in vivo setting is difficult in other model systems (Fig. 2B).

##### The zebrafish exoskeleton allows studying osteoanabolism in an adult setting

As mentioned in the introduction on fish models, zebrafish have a mineralized exoskeleton formed through dermal ossification, consisting of fin rays and scales that harbor osteoblasts and osteoclasts. These fins and scales can fully regenerate ex vivo by making new ECM from de novo differentiated osteoblasts. With the availability of fluorescent reporter lines, this regeneration process can be followed without sacrificing the fish. This allows longitudinal studies of osteoanabolism exceeding osteocatabolism. Omic studies using fin regeneration have mostly focused on the early regeneration stages (Table 2). During its initial stages factors involved in focal adhesion and ECM synthesis pathways are often enriched (Table 2). For example, a proteomic study of early regenerating fins from fish treated with prednisolone showed that proteins involved in ossification (GO:0001503), lysosomal lumen acidification (GO:0007042), ion transport (GO:0006811), the secretory pathway (GO:0045054), and vesicular transport (GO:0016192) were changed.^(^ ^89^ ^)^

The regenerating scale has not been intensively studied, even though scales are abundant, easily accessible, and can be cultured ex vivo in a multiwell setting. They have distinct landmarks from the rims with growing mineralized matrix, housing early osteoblasts, to the center of the scale where late osteoblasts reside. A recent study using bulk RNAseq on regenerating scales showed an enrichment of differentially expressed genes linked to ossification (GO:0001503), hedgehog/smoothened signaling pathway (GO:0007224), insulin‐like growth factor signaling (GO:0048009), and cell adhesion (GO:0007155).^(^ ^84^ ^)^ Moreover, many genes involved in a regenerating scale were enriched for human orthologues that cause monogenic skeletal diseases (eg, *COL1A1‐*, *SP7*‐, *ANO5*‐related osteogenesis imperfecta) and/or are in loci associated with polygenic bone traits (eg BMD, height).^(^ ^84^ ^)^

## Shortening the Diagnostic Timeframe for HBM Disorders in the Future

### The future wave of strategies and technologies to improve HBM gene discovery

Despite the major advances in genomic knowledge and genetic testing, affected individuals often end up in an unsolved or “discovery cohort,” where a novel molecular mechanism is expected to underlie the development of an (un)known HBM phenotype. The remaining challenge in the diagnostics of HBM disorders, therefore, is how best to identify and characterize novel HBM genes, both time‐ and cost‐effectively.

Although most gene discovery to date has arisen from WES, a shift toward WGS will enable researchers to expand beyond exonic variation to assess splicing variants, larger insertions or deletions (InDels), chromosomal rearrangements and repeat expansions (copy number variation), which may uncover novel disease mechanisms. In the case of larger chromosomal abnormalities, alternative detection methods can be used, such as single‐nucleotide polymorphism (SNP) arrays, array comparative genomic hybridization (aCGH), or long‐read sequencing.^(^ ^144^ ^)^ Additionally, mosaic HBM disorders (eg, melorheostosis) may require deep genomic sequencing with read depth of hundreds to thousands, because fewer cells carry the pathogenic variant of interest.^(^ ^145^ ^)^ Defects in gene regulation, as in van Buchem disease cases, are often not yet picked up in a clinical setting. The combined use of WGS and RNAseq (eg, on differentiated iPSCs) could improve the identification of splicing mutations or regulatory DNA mutations (promoter regions, enhancers).

After determining the pathogenicity of variants in accordance with the American College of Medical Genetics and Genomics (ACMG) guidelines, evaluating variants of uncertain significance (VUS), coding or noncoding, for their causality remains challenging.^(^ ^146^ ^)^ Interpretation of substantial amounts of VUS, even after variant filtering, can be extremely time‐consuming. Often, at this stage, larger gene panels are used, for example including all genes listed in the latest ISDS nosology.^(^ ^53^ ^)^ This strategy, however, includes variation in >400 genes related to an immense variety of skeletal phenotypes. Alternatively, VUS linked to the >500 genes or loci listed in genomewide association studies (GWASs) for their association with variance in BMD (as derived from DXA) may be used as a prioritization tool, but often still leaves scientists and clinicians puzzled with a lengthy list.^(^ ^147^, ^148^ ^)^ GWAS‐associated variants also tend to have a small contribution, ie, individually, to the variance in BMD whose biological impact may be different from the processes disturbed by rare variants underlying a HBM disorder. Nevertheless, (few) individuals at the high extreme of the BMD polygenic score distribution can mimic the presence of a monogenic mutation, without harboring one.^(^ ^149^, ^150^ ^)^ Finally, BMD is subject to substantial size artifacts due to its two‐dimensional (2D) nature, so GWAS on BMD will pick up genetic variation in genes affecting growth plate chondrogenesis the same way as those affecting bone mass accrual.

#### Organizing and maximizing rare HBM disease biological sample data

Recent advances in genomic technologies have substantially shortened the diagnostic pathway for rare monogenic HBM disorders, but there is a large amount of data to be managed and analyzed with only a limited number of patients.^(^ ^151^ ^)^ A way to circumvent this bioinformatic challenge is to establish a standardized, and easily accessible registry for HBM patients, clinicians, and basic/translational scientists.^(^ ^152^ ^)^ Similar registries have successfully been set up for other rare bone disorders, such as osteogenesis imperfecta (ROI) (https://oif.org/oiregistry/), Ehlers‐Danlos syndrome (RED) (https://www.ehlers-danlos.com/eds-global-registry/), hypophosphatasia (https://hppregistry.com/), and unifying registries such as the European Registry for rare bone and mineral conditions (https://eurr-bone.com/). An HBM registry could be a pivotal tool to support HBM research and patient management, because the primary aims are collection, analysis, and dissemination of information on a group of people defined by a rare but particular phenotype. To enable data pooling of patients suffering orphan diseases, an input of standardized data is strictly necessary. The use of Human Phenotype Ontology (HPO) terms for phenotypic descriptions (eg, data extracted from X‐rays, bone biopsies) of (un)known HBM disorders, ORPHAcodes and OMIM numbering for referencing HBM disorders and HGVS nomenclature are good examples of standardized approaches to follow. Active inclusion of our classification of HBM genes according to their biological function (Table 1) could be incorporated. Defining a minimum common dataset based on our classification of HBM genes would aid collection of standardized data.

Because HBM cases are few, in‐depth phenotyping is crucial. HBM patients are traditionally screened with X‐ray‐based methods, and phenotyping is based on radiographs and/or by DXA BMD measurements. Besides density measurements, more precise information regarding bone strength, microarchitecture, and fracture risk can be collected by performing high‐resolution peripheral quantitative computed tomography (HR‐pQCT) in parallel. However, its value in routine clinical care of HBM patients must be further explored.^(^ ^153^ ^)^ Phenotypic data derived from serum analysis of bone turnover markers and a transiliac bone biopsy also provide highly valuable insights for HBM diagnostics such as activity and histology of bone cells, structural and dynamic bone properties, matrix composition, and bone mineral density distribution. However, taking a bone biopsy remains an invasive procedure. Alternatively, the use of patient‐derived iPSCs in a clinical setting could be less invasive by differentiating iPSCs into specialized bone cell types using bone matrix scaffolds for laboratory testing (eg, omics, activity, morphology).^(^ ^120^, ^121^ ^)^

Detailed phenotyping, state‐of‐the art genetic screening strategies, and linking genotype–phenotype information to an affected mechanism can make a stark difference in future VUS interpretation for HBM phenotypes. Our classification of HBM genes can be a key tool here (Table 1). Because (sub)groups were labeled with GO accession numbers, this may provide a novel way of interpreting unknown HBM phenotypes or VUS in the clinic based on phenotypic/biological/molecular overlaps within this classification. Especially in multidisciplinary teams, this classification can provide a unified and unifying way to look at novel HBM phenotypes or genes, to ideally shorten the diagnostic timeframe.

#### Artificial intelligence–based technologies to boost HBM diagnostics

Artificial intelligence (AI) algorithms that deploy machine learning and deep neural networks are increasingly used to augment and automate HTS data analysis, eg, improved base calling^(^ ^154^ ^)^ and variant annotation accuracy,^(^ ^155^ ^)^ better detection and prediction of both coding^(^ ^156^, ^157^, ^158^ ^)^ and non‐coding pathogenic variants.^(^ ^159^, ^160^ ^)^ Deep neural networks, or deep learning, builds up from training datasets (eg, images, DNA/amino acid sequences) to perform enhanced predictions on novel unseen data, so that large amounts of data can be used to make objective classifications or predictions, uncovering novel hypothesis‐free (unsupervised) insights that can guide the diagnostic and treatment options of a patient.

AI‐based models have already shown promise in phenotype–genotype mapping, using for example electronic health records and facial images (ie, DeepGestalt, Face‐2‐Gene) for variant prioritization^(^ ^161^, ^162^ ^)^ or by combining WGS data and automated phenotyping, through clinical natural language processing (CNLP) on electronic health records.^(^ ^163^ ^)^ AI‐based tools that combine HTS and phenotypic data (eg, HPO‐terminology) are also already available to generate provisional clinical and molecular diagnoses, such as Moon (https://www.diploid.com/moon).^(^ ^164^ ^)^ Creating AI‐based initiatives, eg, on extraction of data from histological/X‐ray images, may have potential for HBM phenotypic evaluations and genetic testing in the future.

AI also has the potential to aid in VUS interpretation, such as the recently developed deep neural network AlphaFold, that can predict 3D protein structures with atomic accuracy.^(^ ^163^, ^165^ ^)^ For the human proteome, Tunyasuvunakool and colleagues^(^ ^165^ ^)^ expanded its structural coverage by applying AlphaFold at a scale covering almost all human proteins. These predictions are freely available to the community and anticipate that routine large‐scale and high‐accuracy structure prediction will become a valuable tool to address new questions in terms of VUS interpretation (AlphaFold Protein Structure Database, https://alphafold.com/).^(^ ^165^, ^166^ ^)^ Deep learning models have also been trained to further annotate amino acid sequence with protein function throughout the proteome, by using the protein family's database (Pfam; https://www.ebi.ac.uk/interpro/).^(^ ^167^ ^)^ Advances in the coverage of Pfam also suggest that deep learning models will be a core component of future protein annotation tools and VUS interpretation. Finally, interpreting the effects of noncoding variation on gene expression in different cell types remains a major unsolved problem.^(^ ^168^ ^)^ Deep learning models, such as Enformer, can predict gene expression and chromatin states from DNA sequences and may improve the future understanding of transcriptional regulation of HBM disorders (eg, enhancer–promoter interactions).^(^ ^168^ ^)^

## Future Perspectives

**Fig. 3:**
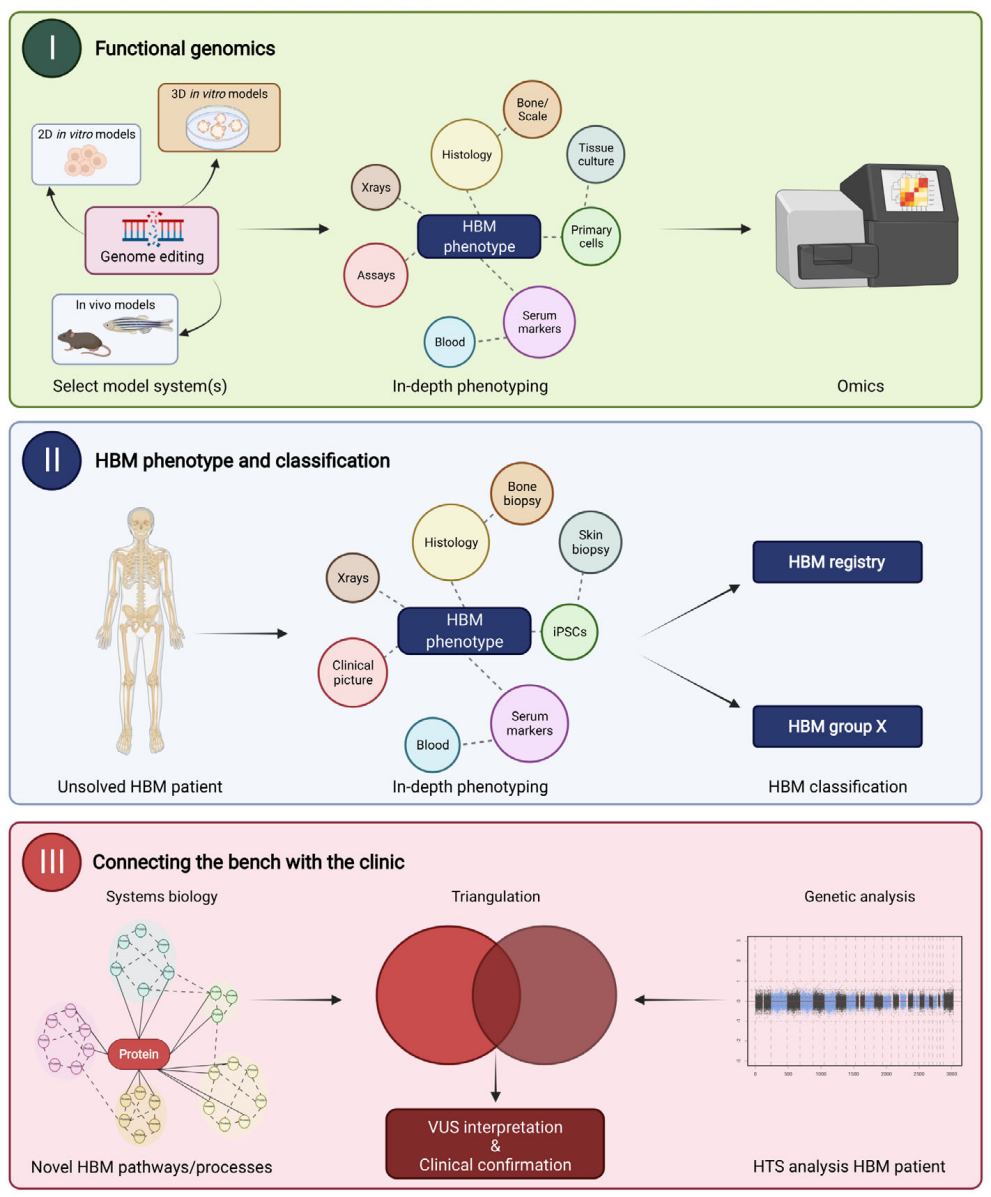
Connecting the bench and clinic with a multidisciplinary reverse genetics pipeline. The reverse genetics pipeline starts with performing functional studies on known HBM genes or risk factors in model systems (panel I). Large‐scale omic approaches allow mapping of disrupted regulatory networks relevant to a specific HBM group. The HBM group classification system allows us to potentially predict which mechanisms may be affected. concurrent phenotyping of genetically unsolved HBM cases may therefore link a phenotype with a pathway or biological process (panel II). By intersecting omic dataset from model systems of that HBM group and with genomic HBM patient data could provide (novel) candidate genes (panel III). HBM = high bone mass; VUS = variants of uncertain significance.

In this review, we collated the available knowledge on HBM, which requires a multifaceted effort. In light of this, we propose triangulation of data generated by basic research from multiple disciplines to improve clinical HBM diagnostics and discover new therapeutic targets for metabolic bone disorders. Our initiative to create a classification system based on biological function may become a valuable tool for researchers and clinicians. A recent screening of pathogenic variants in known HBM genes in an extended HBM cohort identified the genetic cause in only 3% of all cases.^(^ ^5^ ^)^ A significant percentage of the remaining ones are assumed to have a polygenic explanation, but monogenic causes are definitely also missed. These could involve undetected noncoding or copy number variants as well as the involvement of currently unknown modifier genes. We therefore believe that a preferred use of reverse genetic strategies can accelerate novel gene discoveries in the future (Fig. 3). This will be essential to reveal novel HBM genes and their regulatory mechanisms belonging to a given HBM group. The list in Table 1 will undoubtedly continue growing, with generation of novel (sub)groups of the proposed classification.

Compared to other fields of study, the HBM field has not published many studies with omic assessments. Practical factors constitute standing bottlenecks, such as bone tissue being difficult to obtain, taking a long time to grow, and containing a variety of cell types, which all together limit a broader use of omics technologies. As each omic study captures a snapshot of a biological process in time and place, certain considerations should be taken into account when interpreting results: (i) statistical analyses can be challenging as they capture thousands of measurements that can vary greatly between individuals; (ii) the bio‐organization of bone tissue is complex and multilayered (ie, epigenetics, transcriptional and translational inhibition processes, protein dynamics, etc.) resulting in a single omic dataset not necessarily capturing the full biological landscape; and (iii) variation between model organisms, tissues, cell types, bone elements, and state of differentiation could impact the results. Key findings should therefore be replicated with independent experiments in preferably multiple systems that are relevant to HBM biology. Misra and colleagues^(^ ^54^ ^)^ described an integrated multiomics approach to capture causal relationships between “functional potential” and actual “biological activity,” to visualize the actual disease state and provide new HBM candidate genes. This requires an interdisciplinary and multi‐laboratory approach to share knowledge and expertise, especially in the case of rare disorders, to fully define the molecular landscape of HBM.

Similarly for the clinic, the preferred use of WGS for diagnostics of HBM cases will circumvent the inherent blind spot of WES data. Here, our HBM classification system (Table 1) will also aid in the generation of adequate hypotheses to reduce the diagnostic timeframe. Improved, in‐depth phenotyping of HBM patients and setting up a HBM registry are essential as well. New candidate gene discovery can be sped up by triangulating VUS filtered WGS genetic findings with multiomics data sets relevant to a particular HBM group (Fig. 3). Currently, the use of patient iPSCs within the HBM field is still very limited due to cost and complexity of the applied methods, although there is great potential to use it in a clinical setting. Combining patient iPSC‐derived 3D organoid models with other functional genomics tools may also enable a comprehensive translational angle, again allowing novel insights from patient to model system.

An improved diagnosis, classification, and understanding of HBM disorders can impact the treatment and prevention of severe symptoms in affected individuals, often occurring secondary to HBM. For example, affected individuals from HBM group 1A (“Regulation of ossification”—“Regulation of WNT signaling”) often suffer from hearing loss or severe headaches due to progressive cranial hyperostosis and nerve entrapment. Ideally, identification of a variant in a known or novel HBM gene from this particular HBM subgroup could then impact the follow‐up of the affected individual in the clinic to prevent secondary symptoms and improve prognosis to a maximum extent. Deploying a translational pipeline approach that connects the bench with the clinic can also result in the development of targeted and personalized gene‐driven or mutation‐driven therapies, including reprogrammed iPSCs and BMSCs. The need for funding programs that facilitate formation of large consortia allowing for networking of multidisciplinary researchers (eg, COST Actions, European Reference Networks) and undertaking of basic and clinical research (eg, Horizon Europe grants, NIH and other governmental grants) is imperative to attain this goal. Moreover, the use of mRNA‐based therapies could hugely impact HBM disorders, especially for those that are ultrarare. For example, disorders included in HBM group 8 (“Regulation of catalytic activity,” Table 1) can be targeted for enzyme replacement therapy (ERT), which has been used to treat rare and severe conditions such as hypophosphatasia (asfotase alfa; U.S. Food and Drug Administration [FDA] approved [September 2022]), mucopolysaccharidosis type VI (galsulfase; FDA‐approved [September 2022]), and the ABCC6 deficiency (INZ‐701; phase 1/2 clinical trial [September 2022]).^(^ ^169^, ^170^, ^171^ ^)^ Future challenges remain in the development of appropriate delivery methods, especially for notoriously difficult to target cell types, such as osteoblasts. We propose a paradigm shift toward a multidimensional approach based on reverse genetics because this could accelerate the identification of novel therapeutic targets and drugs for HBM disorders that may also benefit rare and common disorders of bone fragility.

## Author Contributions

**Dylan J.M. Bergen:** Conceptualization; funding acquisition; supervision; writing – original draft; writing – review and editing. **Antonio Maurizi:** Conceptualization; funding acquisition; supervision; writing – original draft; writing – review and editing. **Melissa M. Formosa:** Conceptualization; funding acquisition; visualization; writing – original draft; writing – review and editing. **Georgina L.K. McDonald:** Funding acquisition; visualization; writing – original draft; writing – review and editing. **Ahmed El‐Gazzar:** Visualization; writing – review and editing. **Neelam Hassan:** Funding acquisition; writing – original draft. **Maria Luisa Brandi:** Writing – review and editing. **Jose Antonio Riancho:** Writing – review and editing. **FERNANDO RIVADENEIRA:** Funding acquisition; writing – review and editing. **Evangelia Ntzani:** Writing – review and editing. **Emma L Duncan:** Writing – original draft; writing – review and editing. **Celia L Gregson:** Writing – review and editing. **Douglas P. Kiel:** Writing – review and editing. **M. Carola Zillikens:** Writing – review and editing. **Luca Sangiorgi:** Writing – original draft; writing – review and editing. **Wolfgang Högler:** Conceptualization; writing – original draft; writing – review and editing. **Ivan Duran:** Funding acquisition; visualization; writing – original draft; writing – review and editing. **Outi Makitie:** Conceptualization; funding acquisition; writing – original draft; writing – review and editing. **Wim Van Hul:** Conceptualization; funding acquisition; writing – original draft; writing – review and editing. **Gretl Hendrickx:** Conceptualization; supervision; visualization; writing – original draft; writing – review and editing.

## Conflicts of Interest

AEG has received honoraria from Alexion, AstraZeneca Rare Disease. MLB has received honoraria from Amgen, Bruno Farmaceutici, Calcilytix, Kyowa Kirin, UCB. MoLB received grants and/or was a speaker: Abiogen, Alexion, Amgen, Amolyt, Amorphical, Bruno Farmaceutici, CoGeDi, Echolight, Eli Lilly, Enterabio, Gedeon Richter, Italfarmaco, Kyowa Kirin, Menarini, Monte Rosa, SPA, Takada, Theramex, UCB. MLB was a consultant for Aboca, Alexion, Amolyt, Bruno Farmaceutici, Calcilytix, Echolight, Kyowa Kirin, Personal Genomics, UCB. JAR has received research grants, travel grants or lecture fees from Merck, UCB, Amgen, Gedeon Richter, Lilly, Alexion, Takeda and Kyowa Kirin. OM has consulted for or received lecture fees from Kyowa Kirin, BridgeBio, Alexion, Sandoz, and Ultragenyx. ELD has received honoraria for research purposes from Kyowa Kirin and Pharmacosomos, and previously personally from Amgen. WVH received research grants from Roche and Johnson & Johnson and lecture fees or travel grants from Amgen, UCB, and Novartis. All other authors state that they have no conflicts of interest with respect to the submitted manuscript.

### Peer Review

The peer review history for this article is available at https://publons.com/publon/10.1002/jbmr.4715.

## Supporting information


**Table S1** Extended High Bone Mass classification. Excel file containing an extended version of Table 1 with classification of high bone mass groups. A “Read Me” tab is provided with header descriptions and abbreviations.

**Table S2** Transgenic Mouse Models With A High Bone Mass Phenotype. Excel file with two data tabs containing lists of mouse models with high bone mass of disease genes with either a known human high bone mass phenotype or without a described human phenotype. A “Read Me” tab is provided with header descriptions and abbreviations.

## Data Availability

Data sharing is not applicable to this article as no new data were created or analyzed in this study.
